# From Samaritan Fund to Social Service Department

**Published:** 1918-08-03

**Authors:** 


### THE SOCIAL BAROMETER.

### From Samaritan Fund to Social Service Department.

FEW developments of hospital work are more remarkable than that by which the small Samaritan Funds of the hospitals in London have grown into social service departments. The variety and extent of this new phase of hospital usefulness is well seen in the report which Miss Cummins has issued from the lady almoner's department of St. Thomas's Hospital. An out-patient department is a centre of consultation in more senses than one; the patient's home is often a more difficult problem than his disease. This double side of the difficulty explains the extension of organisation, which has far from reached its limit even yet. For those convalescents who do not go to the convalescent home of the hospital the boarding-out system provides, but the cottagers who receive these patients now require nearly twice the payment which used to suffice. This is one of the expenses of the Samaritan Fund. The reverse and more cheerful side of the matter is to be observed in the out-patient department, that barometer of economic pressure among the poor! The out-of-work patients are now unknown, and the family income has more than met the rise in the cost of living. Besides, as a rule, it is now a certainty: the casual is (for the moment) a man of the past; and once the Army or the pension committee, or the munition authority begins to pay, it pays with commendable punctuality. Against this must be set the deterioration of the houses of the working classes in South London. If it were not that they are inhabited, the Germans would do us a good turn to blow them up with dynamite. Miss Cummins' description of them deserves to be quoted unabridged:

With a few notable exceptions, such as occur in the Duchy of Cornwall estate, the houses from which St. Thomas's Hospital draws many of its patients are absolutely unfitted for human habitation. If this were due to the absence of labour for necessary repairs, it would probably be unwise to dwell on the fact, but this is not the case. Within a few minutes' walk of the hospital there are streets of small houses that are an absolute disgrace, and that ought not to exist.

Much attention has been drawn to the deplorable sanitary conditions to which our prisoners have been subjected in Germany, and rightly so; but at times one is tempted to ask why the nation has so long allowed many of the inhabitants of its richest city to live in some of the overcrowded insanitary dwellings that exist near the hospital. Some of the rooms in which St. Thomas's patients are found would be unfit even for cattle, and yet human beings live in them and rear their children in such conditions. When it is remembered that there is hardly a dwelling in Lambeth with any provision whatever for keeping food, that shelves and cupboards do not exist, that nearly all lavatories receive their sole light from the door, which sometimes opens on public stairs or dark passages, and often all water has to be carried up many flights of stairs, that baths are non-existent, that downstairs storage for perambulators is impossible, that even model buildings will be found mostly so constructed that the rooms are long and narrow, with window opposite door . . . one is inclined to long for some English modification of the American lady with the axe who smashed saloon fittings to draw attention to their evils.

If scarcity of labour does not explain the dilapidation into which these miserable hovels have fallen, what is the explanation?

It is not perhaps surprising, then, that Miss Cummins has found the district nurse to be a proved success; to have reduced the number of cases of broncho-pneumonia consequent upon an attack of bronchitis, to have diminished the number of serious cases of infantile diarrhœa. Still, children's medical beds are needed in greater numbers. It is recorded that cleanliness is more popular than it used to be, and that the welcome given by mothers to an explanation of the cause of scabies and kindred troubles is taken to prove that the patients themselves are not to blame. Since we have already been acquainted with their homes, it is clear that the responsibility largely rests with the landlord, who is as surely spreading disease as if he inoculated the patients with it. The names of the owners of this property should be published, even if they include the Ecclesiastical Commission itself.

It is satisfactory to hear that "it is impossible to bring a child regularly to the Baby Clinic without getting on very friendly terms with the almoner's department." The mothers, we learn, recruit new patients for the children's surgical department, and this further testimony reminds us that a voluntary hospital is protected from the officiousness of Charity Organisation-mongering, since it comes to the helpless with something to give, and not to find an excuse for taking something away.

The venereal disease department, again, is the test of the value of legislation, and it is pleasant to know that letters signed by the medical officer of the department are sent to patients who are over-due, and this reminder of the value of the treatment is often successful. To meet the need of those who attended late in the evening and were in a highly infectious state, for whom no beds were available, while "lack of immediate means would force them to earn their night's lodging in a manner disastrous to the public health," three boroughs have been persuaded to admit patients to their infirmaries, where they are sent direct, and usually in a cab. In this way the women's fear of the relieving officer has been overcome. In the case of married women, and with their consent, when advisable a letter is forwarded to the husband recommending him to seek advice, at the hospital or the nearest place, and this is generally done except when the nearest place is the office of the regimental surgeon.

The disabled soldier is a present problem. The discharged will be another in due time. For the former a special register has been provided. For the latter, in the case of "contacts" alone, much work will have to be done. The vista opened by the Samaritan Fund is a wide one. But the interest is only equalled by the importance of the work.

